# Mortality and Morbidity in Infants <34 Weeks' Gestation in 25 NICUs in China: A Prospective Cohort Study

**DOI:** 10.3389/fped.2020.00033

**Published:** 2020-02-13

**Authors:** Siyuan Jiang, Weili Yan, Shujuan Li, Lan Zhang, Yi Zhang, Prakesh S. Shah, Vibhuti Shah, Shoo K. Lee, Yi Yang, Yun Cao

**Affiliations:** ^1^Department of Neonatology, Children's Hospital of Fudan University, Shanghai, China; ^2^Department of Clinical Epidemiology, Children's Hospital of Fudan University, Shanghai, China; ^3^Maternal-Infant Care Research Centre and Department of Pediatrics Mount Sinai Hospital, Toronto, ON, Canada; ^4^Department of Pediatrics, University of Toronto, Toronto, ON, Canada; ^5^Department of Obstetrics and Gynecology and Dalla Lana School of Public Health, University of Toronto, Toronto, ON, Canada; ^6^NHC Key Laboratory of Neonatal Diseases (Fudan University), Children's Hospital of Fudan University, Shanghai, China

**Keywords:** outcome, mortality, morbidity, preterm infants, China

## Abstract

**Objectives:** To describe the rates and variability of mortality and morbidity of preterm infants born in China.

**Methods:** This prospective cohort study included infants born at <34 weeks' gestation and admitted to 25 NICUs within 7 days of birth between May 1st, 2015 and April 30th, 2016. Infants were followed until death or NICU discharge. The primary outcome was a composite of mortality or any major morbidity (sepsis, necrotizing enterocolitis, intraventricular/periventricular leukomalacia, retinopathy of prematurity, and bronchopulmonary dysplasia) in infants who received complete care following medical advice. Secondary outcomes included rate of discharge against medical advice, mortality and individual morbidities.

**Results:** Of the 8,065 infants, 6,852 (85%) received complete care and 1,213 (15%) were discharged against medical advice. Among infants who received complete care, the rate of the composite outcome was 27% (1,827/6,852), mortality 4% (248/6,852), sepsis 14% (990/6,852), necrotizing enterocolitis 3% (191/6,550), intraventricular hemorrhage/periventricular leukomalacia 7% (422/6,307), retinopathy of prematurity 2% (67/3,349), and bronchopulmonary dysplasia 9% (616/6,852). There were significant variations between NICUs for all outcomes.

**Conclusions:** Discharged against medical advice, mortality, and morbidity rates for preterm infants <34 weeks' gestation are high in China with significant variations between NICUs.

## Introduction

Preterm birth (<37 weeks' gestation) is the leading cause of childhood mortality in China (1–4). Infants born preterm are also at high risk of developing morbidities such as sepsis, necrotizing enterocolitis (NEC), intraventricular hemorrhage (IVH), periventricular leukomalacia (PVL), retinopathy of prematurity (ROP), and bronchopulmonary dysplasia (BPD) (5). With the advancement of perinatal-neonatal medicine, the number of preterm infants cared for in Chinese NICUs has increased (6). Collecting comprehensive, high-quality data on mortality and morbidity in infants born preterm is crucial to understanding trends, identifying opportunities for quality improvement, and ultimately improving infant outcomes (7). However, reports of mortality and morbidity rates for preterm infants in China are limited to small studies using varying definitions (8–10).

Our study aimed to use a newly established neonatal database to create a comprehensive report of mortality and morbidity for infants born at <34 weeks' gestation admitted to 25 NICUs in China as well as the outcome variation between NICUs. Our results provide baseline data for epidemiological and quality improvement studies.

## Materials and Methods

### Study Design and Population

All infants born at <34 weeks' gestation between May 1st, 2015 and April 30th, 2016 and admitted to participating NICUs within 7 days of birth were eligible for this prospective cohort study. Exclusion criteria were stillborn and delivery room deaths. The endpoint of the study was death or discharge from NICU.

### Setting

**Figure 1:**
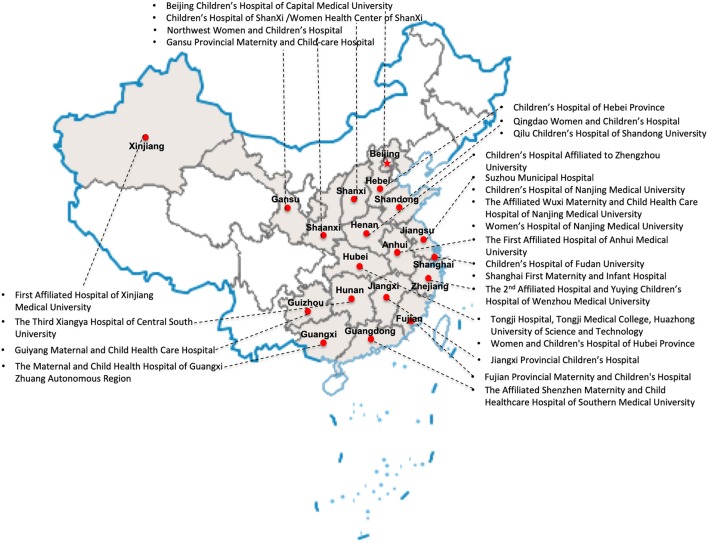
Locations of participating hospitals.

Twenty-five tertiary hospitals from 19 provinces in China (Figure 1) able to care for infants born at <28 weeks' gestation or weighing <1,000 grams at birth participated in this study. Seventeen hospitals were national or provincial neonatal referral centers, and eight were regional referral centers in metropolitan cities (Supplementary Table 1).

### Data Collection

The database was developed in collaboration with the Canadian Neonatal Network (CNN) for a cluster randomized controlled study entitled “Reduction of Infection in Neonatal Intensive Care Units using the Evidence-based Practice for Improving Quality (EPIQ)” (REIN-EPIQ study, clinicaltrials.gov #NCT02600195). Before initiating the intervention, clinical data of eligible patients admitted to NICUs between January 1, 2015 and December 31, 2016 were collected as background data, and these patients comprise the current study population.

All data collection followed standard operations and definitions developed in collaboration with CNN (11). Trained data abstractors prospectively collected infant information at each NICU. Data were electronically transmitted monthly to the Coordinating Center located in the Children's Hospital of Fudan University and de-identified prior to analysis. The Coordinating Center checked data for quality and completeness, generated detailed reports for each NICU, and conducted center visits and audits of data collection at each NICU at least once during the study period.

### Variable Definitions

Gestational age was determined using the hierarchy of best obstetric estimate based on prenatal ultrasound, menstrual history, obstetric examination, or all three. If the obstetric estimate was not available or was different from the postnatal pediatric estimate of gestation by more than 2 weeks, the gestational age was estimated using the Ballard Score (12). Small for gestational age (SGA) was defined as birth weight <10th percentile for the gestational age according to the Chinese neonatal birth weight values (13). Prenatal care was defined as at least one pregnancy-related hospital visit during pregnancy. Antenatal corticosteroid treatment was defined as partial or complete course of antenatal corticosteroids prior to birth. Transport Risk Index of Physiologic Stability (TRIPS) score (14, 15) was used as an illness severity score on NICU admission.

### Outcomes

The primary outcome was a composite of mortality or one of five major morbidities including sepsis, NEC, IVH, PVL, ROP, or BPD in infants who received complete care. Complete care infants were infants who received medical care in NICUs until treating physicians recommended discharge or died. were treated following medical advice. The secondary outcomes were rate of discharge against medical advice (DAMA), mortality and each of the morbidities individually. Discharge against medical advice was defined as when parents terminated treatment before the treating physicians recommended discharge according to discharge criteria in participating hospitals. Sepsis included both culture-proven sepsis and clinical sepsis. Culture-proven sepsis was diagnosed according to Stoll et al. (16). Clinical sepsis was diagnosed when blood culture was negative and all of the following three criteria were fulfilled: (1) two or more infection-related clinical manifestations; (2) abnormal white blood cell count, C-reactive protein level, or procalcitonin level; and (3) antibiotics for ≥5 days. NEC was defined as ≥ stage 2 according to Bell's criteria (17, 18). IVH was defined as ≥ grade 3 according to Papile's criteria (19). PVL was defined as the presence of periventricular cysts on cranial ultrasound or cranial MRI scans. ROP was defined as ≥ stage 3 according to the International Classification of ROP (20). BPD was defined as mechanical ventilation or oxygen dependency at 36 weeks' postmenstrual age or discharge (21).

Rate of DAMA is an important secondary outcome because it may significantly influence the rates of other outcomes (8, 10, 22–24). Once infants are DAMA, we were unable to determine if they survived because of logistical constraints; however, we used pre-defined criteria to predict the likelihood of death for these infants. If infants DAMA required invasive or non-invasive mechanical ventilation, inotropes infusion, or total parenteral nutrition (no enteral feeds initiated) on the day of discharge, we predicted that they would not survive without support. Our primary and secondary outcomes were only calculated among the complete care infants because we were unable to determine the morbidities of DAMA infants.

### Statistical Analysis

Maternal and neonatal characteristics were compared using Student's *t*-tests for continuous variables and Chi-square tests for categorical variables. A multivariable logistic regression model was used to calculate adjusted odds ratios (aOR) with a 95% confidence interval (CI) of each outcome among participating NICUs. We controlled for sex, gestational age, SGA, Apgar score <3 at 5 min, TRIPS score on admission, inborn, cesarean, maternal hypertension, maternal diabetes, and antenatal steroids. A subgroup analysis of inborn infant was done using the same methods of analysis above. A two-sided *P* < 0.05 was used to determine statistical significance. Statistical analysis was performed using Stata 13.1 (StataCorp, 2013, College Station, Texas, USA).

## Results

### Infant Characteristics

A total of 8,067 eligible infants were admitted during the study period. Two infants were excluded for missing discharge data. The median number of infants enrolled in each participating NICU was 321 (range 72–508). A majority of infants were born at ≥30 weeks' gestation and ≥1,500 grams birth weight (Table 1).

**Table 1:** Characteristics of mothers and infants born at <34 weeks' gestation.

**Characteristics**	**All infants**	**Complete care infants**	**DAMA infants**	***P*-value**
Number of infants	8,065	6,852	1,213	–
**Maternal characteristics**				
Prenatal care, *n*/*N* (%)	7,782/8,008 (97.2)	6,710/6,802 (98.6)	1,172/1,206 (97.2)	<0.001
Maternal hypertension, *n*/*N* (%)	1,290/7,940 (16.2)	1,104/6,751 (16.4)	186/1,189 (15.6)	0.541
Maternal diabetes, *n*/*N* (%)	843/7,936 (10.6)	752/6,748 (11.1)	91/1,188 (7.7)	<0.001
Antenatal steroids, *n*/*N* (%)	4,860/7,635 (63.7)	4,232/6,498 (65.1)	628/1,137 (55.2)	<0.001
Primigravida, *n*/*N* (%)	3,108/8,059 (38.6)	2,678/6,846 (39.1)	430/1,213 (35.4)	0.017
Cesarean section, *n*/*N* (%)	4,186/8,063 (51.9)	3,723/6,851 (54.3)	463/1,212 (38.2)	<0.001
**Infant characteristics**				
Gestational age (weeks), mean (SD)	31.4 (2.0)	31.5 (1.9)	30.4 (2.3)	<0.001
<26^+0^ weeks', *n*/*N* (%)	97/8,065 (1.2)	59/6,852 (0.9)	38/1,213 (3.1)	
26^+0^–26^+6^ weeks', *n*/*N* (%)	141/8,065 (1.7)	97/6,852 (1.4)	44/1,213 (3.6)	
27^+0^–27^+6^ weeks', *n*/*N* (%)	257/8,065 (3.2)	164/6,852 (2.4)	93/1,213 (7.7)	
28^+0^–28^+6^ weeks', *n*/*N* (%)	543/8,065 (6.7)	411/6,852 (6.0)	132/1,213 (10.9)	
29^+0^–29^+6^ weeks', *n*/*N* (%)	744/8,065 (9.2)	588/6,852 (8.6)	156/1,213 (12.9)	
30^+0^–30^+6^ weeks', *n*/*N* (%)	950/8,065 (11.8)	789/6,852 (11.5)	161/1,213 (13.3)	
31^+0^–31^+6^ weeks', *n*/*N* (%)	1,386/8,065 (17.2)	1,197/6,852 (17.5)	189/1,213 (15.6)	
32^+0^–32^+6^ weeks', *n*/*N* (%)	1,822/8,065 (22.6)	1,615/6,852 (23.6)	207/1,213 (17.1)	
33^+0^–33^+6^ weeks', *n*/*N* (%)	2,125/8,065 (26.3)	1,932/6,852 (28.2)	193/1,213 (15.9)	
Birth weight (grams), mean (SD)	1636 (414)	1,668 (406)	1,448 (408)	<0.001
<750 grams, *n*/*N* (%)	66/8,065 (0.8)	43/6,852 (0.6)	23/1,213(1.9)	
750–999 grams, *n*/*N* (%)	380/8,065 (4.7)	261/36,852 (3.8)	119/1,213 (9.8)	
1,000–1,249 grams, *n*/*N* (%)	1,010/8,065 (12.5)	744/ 6,852 (10.9)	266/1,213 (21.9)	
1,250–1,499 grams, *n*/*N* (%)	1,500/8,065 (18.6)	1,240/6,852 (18.1)	260/1,213(21.4)	
1,500–1,999 grams, *n*/*N* (%)	3,468/8,065 (43.0)	3,056/6,852 (44.6)	412/1,213 (34.0)	
≥ 2,000 grams, *n*/*N* (%)	1,641/8,065 (20.3)	1,508/6,852 (22.0)	133/1,213 (11.0)	
Male, *n*/*N* (%)	4,721/8,065 (58.5)	4,032/6,852 (58.8)	689/1,213 (56.8)	0.183
SGA, *n*/*N* (%)	995/8,065 (12.3)	805/6,852 (11.7)	190/1,213 (15.7)	<0.001
1-min Apgar ≤ 3, *n*/*N* (%)	396/7,713 (5.1)	277/6,602 (4.2)	119/1,111 (10.7)	<0.001
5-min Apgar ≤ 3, *n*/*N* (%)	72/7,244 (1.0)	49/6,227 (0.8)	23/1,017 (2.3)	<0.001
TRIPS score, median (IQR)	12 (6–19)	11 (6–19)	18 (8–24)	<0.001
Inborn, *n*/*N* (%)	5,517/8,065 (68.4)	4,735/6,852 (69.1)	782/1,213 (64.5)	0.001

*NICUs, neonatal intensive care units; DAMA, discharged against medical advice; SD, standard deviation; SGA, small for gestational age; TRIPS, Transport Risk Index of Physiologic Stability score; IQR, interquartile range*.

### Discharge Against Medical Advice

Of 8,065 infants, 15% (1,213) were DAMA (Table 1). Infants who were DAMA were born at a younger mean gestational age and lower mean birth weight; more likely to be SGA, have lower Apgar scores, and higher illness severity on admission; and less likely to be inborn, receive antenatal steroids, and be born by cesarean section than complete care infants (Table 1). At the time of discharge, 32.8% (399/1,213) of infants DAMA were on invasive mechanical ventilation, 24.5% (297/1,213) were on non-invasive ventilation, 15.7% (190/1,213) were on inotropes, and 41.2% (500/1,213) were receiving total parenteral nutrition. Based on the infant characteristics at discharge, we predicted an estimated 64.9% (788/1,213) infants died after NICU-discharge.

### Outcomes

A total of 6,852 infants received complete care in NICUs. The rate of composite outcome was 26.7% (1,827/6,852) among complete care infants (Table 2). Three quarters (240/320) of infants <28 weeks' gestation died or had at least one morbidity during hospitalization. The rate of composite outcome decreased as gestational age increased; however, the rate of composite outcome was still 35.6% (1,062/2,985) in infants born at 28–31weeks' and 14.8% (525/3,547) in infants born at 32–33 weeks' gestation.

**Table 2:** Outcome rates of infants born at <34 weeks' gestation and receiving complete care by gestational age.

**Outcomes**	** <26^**0**^ weeks'**	**26^**0**^–27^**6**^ weeks'**	**28^**0**^–31^**6**^ weeks'**	**32^**0**^–33^**6**^ weeks'**	**Total**
Composite outcome, *n*/*N* (%)	52/59 (88.1)	188/261 (72.0)	1062/2,985 (35.6)	525/3,547 (14.8)	1,827/6,852 (26.7)
Mortality, *n*/*N* (%)	31/59 (52.5)	54/261 (20.7)	129/2,985 (4.3)	34/3,547 (1.0)	248/6,852 (3.6)
Sepsis, *n*/*N* (%)	17/59 (28.8)	95/261 (36.4)	612/2,985 (20.5)	266/3,547 (7.5)	990/6,852 (14.4)
NEC^a^, *n*/*N* (%)	4/37 (10.8)	17/230 (7.4)	113/2,895 (3.9)	57/3,388 (1.7)	191/6,550 (2.9)
IVH or PVL^b^, *n*/*N* (%)	10 /38 (26.3)	47/235 (20.0)	236/2,810 (8.4)	129/3,224 (4.0)	422/6,307 (6.7)
ROP^c^, *n*/*N* (%)	9/24 (37.5)	20/191 (10.5)	31/1,987 (1.6)	7/1,147 (0.6)	67/3,349 (2.0)
BPD, *n*/*N* (%)	45/59 (76.3)	99/261 (37.9)	340/2,985 (11.4)	132/3,547 (3.7)	616/6,852 (9.0)

a *Incidence of NEC ≥ stage 2 = number of infants with NEC ≥stage 2/ number of infants survived more than 72 h*.

b *Incidence of IVH ≥ grade 3 or PVL = number of infants with IVH ≥grade 3 or PVL/ number of infants with neuroimaging results*.

c *Incidence of ROP ≥ stage 3 = number of infants with ROP ≥stage 3/number of infants with eye examinations in NICU*.

*DAMA, infants discharged against medical advice; BPD, bronchopulmonary dysplasia; NEC, necrotizing enterocolitis; IVH, intraventricular hemorrhage; PVL, periventricular leucomalacia; ROP, retinopathy of prematurity*.

Mortality among infants with complete care was as high as 52.5% (31/59) for infants born at <26 weeks' and 20.7% (54/261) for infants born at 26–27 weeks' gestation (Table 2). The mortality rate decreased dramatically for infants born at ≥28 weeks' gestation. Sepsis was the most common morbidity followed by BPD then severe IVH or PVL.

The results of subgroup analysis of outcomes among inborn infants received complete care were shown in Supplementary Tables 2, 3.

### Variation in Outcomes

**Figure 2:**
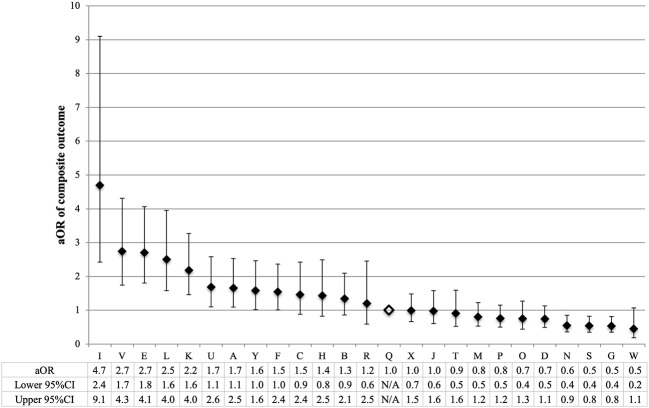
Variation by NICU of composite outcome of infants born at <34 weeks' gestation who received complete care. NICU Q (open diamond) was the reference center in all regression models. To calculate the adjusted odds ratio (aOR), the adjusted covariates were sex, gestational age, small for gestational age, Apgar score <3 at 5 min, TRIPS score on admission, inborn, cesarean, maternal hypertension, maternal diabetes, and antenatal steroids. Error bars indicate 95% confidence intervals (CI).

**Figure 3:**
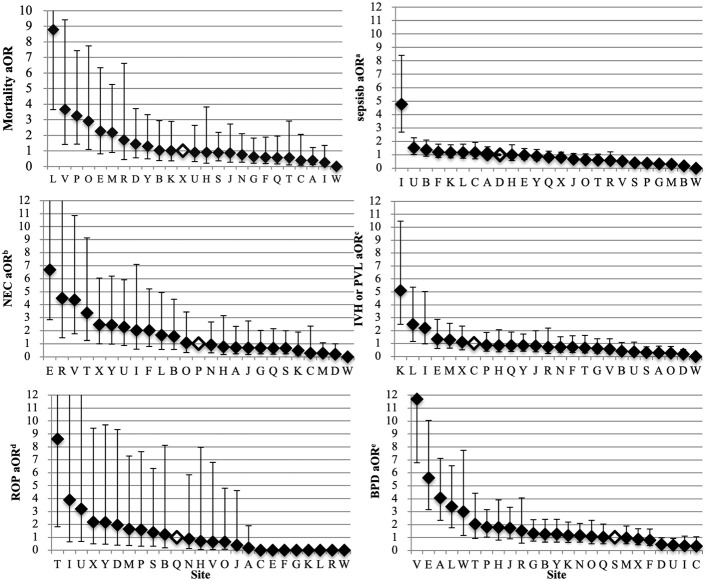
Variation by NICU of mortality and morbidity of infants born at <34 weeks' gestation who received complete care. The NICU with the median outcome rate was used as the reference site for the multi-regression model (open diamond). The adjusted covariates were sex, gestational age, small for gestational age, Apgar score <3 at 5 min, TRIPS score on admission, inborn, cesarean, maternal hypertension, maternal diabetes and antenatal steroids. Error bars indicate 95% confidence intervals. NICUs, neonatal intensive care units; aOR, adjusted odds ratios; NEC, necrotizing enterocolitis; IVH, intraventricular hemorrhage; PVL, periventricular leukomalacia; ROP, retinopathy of prematurity; BPD, bronchopulmonary dysplasia. ^a^Sepsis included both culture-proven sepsis and clinical sepsis. ^b^Included all infants who survived 72 h. NEC was defined as NEC ≥ stage 2 according to Bell's criteria. ^c^Included all infants with neuroimaging results. IVH was defined as IVH ≥ grade 3 according to Papile et al. or PVL. ^d^Included all infants with eye examinations. ROP was defined as ≥ stage 3 according to international classification of ROP. ^e^BPD was defined as requiring mechanical ventilation or oxygen dependency at 36 weeks' postmenstrual age or discharge.

The odds of composite outcome for complete care infants varied across all participating centers (Figure 2). Nine centers had significantly higher odds and three centers had lower odds of the composite outcome than the reference center (center Q). The odds of individual outcomes for complete care infants varied substantially between and within NICUs (Figure 3). For example, NICU V had high odds of mortality and NEC, but their odds of IVH were below the median.

## Discussion

Our study is the largest multicenter study describing preterm infant outcomes in China. We identified high rates of DAMA among preterm infants. For infants receiving complete care in the NICU, we identified rates of mortality and major morbidities that varied between NICUs across China. These data are useful for exploring ways of improving quality of care and health care delivery.

The gestational age distribution of preterm infants admitted to Chinese NICUs is strikingly different from that of developed countries. Compared to Canadian NICUs, Chinese NICUs admitted a higher proportion of infants born at 32–33 weeks' gestation (49% in China vs. 34% in Canada) and a lower proportion of infants born at <29 weeks' gestation (13% in China vs. 32% in Canada) and <26 weeks' gestation (9% in China vs. 34% in Canada) (25). China does not stipulate the lower limit of gestational age at which resuscitation should be offered; however, resuscitation and active care are less routinely offered at 22–26 weeks' gestation. Therefore, China may admit fewer extremely preterm infants (<28 weeks' gestation) than other countries because of differences in the gestational age of viability. The organization of neonatal care provision, such as the gestational age limitation for tertiary care, may differ between countries. Besides, delivery room deaths were excluded in our study and outborn infants were included in our study population, so selective admission and referral may bias the gestational age distribution from the birth population. Further population-based study is needed to investigate the gestational age distribution of all live births and rates of outcomes of preterm infants, especially those smallest ones, in relation with the number of live births. Our data may also serve as a historical reference for the longitudinal trend of gestational age or birth weight distribution of preterm infants receiving adequate care in Chinese NICUs.

One discrepancy between Chinese data and other countries is that infants are DAMA. Previous studies showed preterm infant mortality rates ranging from 53 to 92% as a result of DAMA (8, 10, 22–24), which was reported to be as high as 21% (23). Parent's inability to afford the high cost of NICU care and concern about poor long-term prognosis might be the primary reasons for terminating treatment (10, 22–24). We report that 15% of infants born at <34 weeks' gestation were discharged against medical advice, and the rate decreased with increasing gestational age. However, 13% of infants born at >29 weeks' gestation were still discharged against medical advice. Given the low mortality rate for infants receiving complete care and the large number of infants born at >29 weeks' gestation, efforts to reduce the rate of discharge against medical advice among these infants are urgent. Ideally, the outcome of infants discharged against medical advice should be determined by follow up visits. Previous studies reported a high rate of infants discharged against medical advice in China; however, none reported their methods of follow-up or estimation of outcomes (8, 10, 22–24). We also did not manage to follow up the infants discharged against medical advice. However, our detailed data collection prior to infants' discharge and pre-defined definition allowed us to predict outcomes for these infants. We recognize the limitations to our approach, and it may underestimate mortality. Suboptimal temperature control, inappropriate feeding practices, infection, and other conditions after discharge decrease the chance of survival for discharged infants. Therefore, though our estimated mortality rate was 65% after discharge against medical advice, the actual mortality may be even higher. Further investigations are urgently needed to identify specific reasons for DAMA and strategies to guarantee more preterm infants receive appropriate care.

Our calculated mortality rates for infants who received complete care should be interpreted with caution. Infants who received complete care were born at later gestational ages, less sick on admission, and delivered in-hospital more often than DAMA infants. Therefore, the in-hospital mortality rate may be lower than it would be if all infants received complete care. Even underestimated, the in-hospital mortality rates in our study remain higher than those of Canada, especially at lower gestational ages (25). For example, in 2015 the mortality rate in China was 53% for infants born at <26 weeks' and 21% for infants born at 26–27 weeks' gestation; whereas, the mortality rate in Canada was 33% for infants born at <26 weeks' and 15% for infants born at 26–27 weeks' gestation.

One quarter of preterm infants born at <34 weeks' gestation who received complete care died or experienced one or more serious morbidity, similar to rates from NICHD around 10 years ago (2003–2007) (26). Since then the outcomes of preterm infants in the US improved because of quality improvement efforts (5, 27). Currently collaborative quality improvement initiatives have been initiated to reduce infections in Chinese NICUs (28). More quality improvement efforts should focus on mortality and other morbidities such as IVH or BPD. Infants ≥28 weeks' gestation comprised 94% of all infants born at <34 weeks' gestation and had a relatively low mortality rate if they received complete care. However, the rate of composite outcome among these infants was as high as 24%. Methods to improve the quality of survival of preterm infants should be considered in China.

The demographic profile of patients admitted to Chinese NICUs reveals several points of interest. One third of preterm infants were born outside of a tertiary care setting, which is associated with increased adverse outcomes (29). Also, only 63% of infants born at <34 weeks' gestation received antenatal steroids even though it is one of the most important interventions for the prevention of death and complications for preterm infants (30). Optimized care for mothers with threatened preterm birth and prompt *in-utero* transport when feasible should be considered to improve infant outcomes.

Our study is the first to report variations in outcomes between Chinese NICUs. Wide variations in mortality and serious morbidities between NICUs exist even though the participating NICUs were all tertiary units in metropolitan cities. These results are similar to what was reported by other networks, many of which have reduced variability between NICUs using collaborative networking and quality improvement initiatives (27, 31, 32).

Our study has several strengths. The large prospective cohort including infants of <34 weeks' gestation admitted to NICUs in China enabled us to report the latest infant outcomes by gestational age without selection bias. Multiple measures were applied to ensure the quality of data collection. All participating NICUs were large tertiary centers representing the highest level of neonatal care making the infant outcomes synthesizable and comparable. The definitions of outcomes were standardized to enable comparisons between our results and well-recognized neonatal networks from around the world.

However, there are also limitations to our study. First, the findings are limited to preterm infants admitted to tertiary NICUs in metropolitan cities; they do not reflect the outcomes of infants born in rural areas or smaller cities. Second, this is a hospital-based study instead of population-based study; our results do not represent the outcomes of all live-born preterm infants in China, especially when a substantial number of extremely or very preterm infants are not admitted to NICUs. We also did not include delivery deaths in our analysis. Third, we could not calculate an accurate mortality rate for all infants because of the high proportion of DAMA infants. We used a reasonable algorithm to predict the outcome of these infants, but they should be followed after discharge to determine their actual survival status.

## Conclusion

Our study provides a detailed account of outcomes for infants born at <34 weeks' gestation and admitted to participating NICUs in China. Mortality and morbidity of preterm infants remain high in China with significant variations among NICUs. Many preterm infants do not receive complete care in Chinese NICUs. Further quality improvement initiatives are needed to ensure infants receive appropriate care and reduce adverse neonatal outcomes in China. Further population-based investigations are also needed to look into outcomes of all live births of preterm infants.

## Data Availability Statement

The datasets for this article are not publicly available because: part of the data is included in other article under preparation. Requests to access the datasets should be directed to Yun Cao, yuncao@fudan.edu.cn.

## Ethics Statement

The study was approved by the Ethics Committee of the Children's Hospital of Fudan University, which was recognized by all participating centers. Waiver of informed consent was approved by the ethics committee. All participating centers signed data usage agreements with Children's Hospital of Fudan University.

## Author Contributions

SJ, SKL, and YC conceptualized and designed the study. SJ, SL, YZ, and LZ acquired the data. SJ, WY, and YZ analyzed the data. SJ, PS, VS, YY, SKL, and YC interpreted the results. SJ drafted the manuscript. All authors reviewed and revised the manuscript for important intellectual content and approved the final manuscript as submitted and agree to be accountable for all aspects of the work.

### Conflict of Interest

The authors declare that the research was conducted in the absence of any commercial or financial relationships that could be construed as a potential conflict of interest.
