# Supplementary material for: Mortality and Morbidity in Infants <34 Weeks' Gestation in 25 NICUs in China: A Prospective Cohort Study
Source: Front Pediatr. 2020 Feb 13;8:33. doi: 10.3389/fped.2020.00033 (PMC7031923; doi:10.3389/fped.2020.00033)
Supplement: Supplementary file 1 [file Table_1.docx]

# Supplementary Table 1. Baseline characteristics of participating hospitals

| Hospital | Type of Hospital | Inborn/Outborn | Delivery/ Year | Teaching Hospital | NICU Beds | Intermediate/ Continuing Care Beds | Neonatologists | Nurses | Transport Team | General Surgery | PDA Ligation | Cardiac Surgery | ECMO | MRI |
| --- | --- | --- | --- | --- | --- | --- | --- | --- | --- | --- | --- | --- | --- | --- |
| Shanghai First Maternity and Infant Hospital | Perinatal center | I | 30 000 | Y | 38 | 70 | 30 | 79 | Y | N | N | N | N | Y |
| The Maternal and Child Health Hospital of Guangxi Zhuang Autonomous Region | Perinatal center | I/O | 16 000 | N | 80 | 80 | 31 | 136 | Y | Y | Y | Y | N | N |
| Children’s Hospital of Hebei Province | Children's hospital | I/O | 1600 | Y | 27 | 120 | 22 | 71 | Y | Y | Y | Y | Y | Y |
| Northwest Women and Children’s Hospital | Perinatal center | I/O | 24 000 | Y | 50 | 150 | 37 | 138 | Y | Y | Y | Y | Y | Y |
| Gansu Provincial Maternity and Child-care Hospital | Perinatal center | I/O | 21 000 | N | 70 | 150 | 9 | 101 | Y | Y | Y | Y | Y | Y |
| Qingdao Women and Children’s Hospital | Perinatal center | I/O | 14 000 | Y | 80 | 50 | 13 | 75 | N | Y | Y | Y | Y | Y |
| Obstetrics and Gynecology Hospital Affiliated to Nanjing Medical University | Perinatal center | I/O | 24 626 | Y | 60 | 70 | 27 | 55 | Y | N | Y | N | N | Y |
| Qilu Children’s Hospital of Shandong University | Children' hospital | O | — | Y | 120 | 46 | 32 | 90 | Y | Y | Y | Y | N | Y |
| Children’s Hospital Affiliated to Zhengzhou University | Children' hospital | O | － | Y | 130 | 100 | 35 | 160 | Y | Y | Y | Y | Y | Y |
| The Affiliated Wuxi Maternity and Child Health Care Hospital of Nanjing Medical University | Perinatal center | I/O | 18 000 | Y | 20 | 40 | 20 | 48 | N | N | N | N | N | N |
| Tongji Hospital,Tongji Medical College,Huazhong University of Scinece and Technology | General hospital | I/O | 6000 | Y | 30 | 25 | 8 | 55 | Y | Y | Y | Y | N | Y |
| First Affiliated Hospital of Xinjiang Medical University | General hospital | I/O | 6000 | Y | 30 | 15 | 9 | 34 | Y | Y | Y | Y | N | Y |
| Children’s Hospital of ShanXi / Wonwen Health Center of ShanXi | Perinatal center | I/O | 8500 | N | 96 | 56 | 12 | 104 | Y | Y | Y | Y | N | Y |
| Women and Children's Hospital of Hubei Province | Perinatal center | I/O | 24 000 | N | 53 | 150 | 31 | 154 | Y | Y | Y | Y | N | Y |
| Children’s Hospital of Nanjing Medical University | Children' hospital | O | — | Y | 30 | 98 | 32 | 103 | Y | Y | Y | Y | Y | Y |
| Fujian Provinvial Maternity and Children's Hospital | Perinatal center | I/O | 16 000 | Y | 30 | 110 | 16 | 45 | Y | Y | Y | Y | Y | Y |
| The 2^nd^ Affiliated Hospital and Yuying Children’s Hospital of Wenzhou Medical University | General hospital | I/O | 11 820 | Y | 65 | 65 | 34 | 108 | Y | Y | Y | Y | Y | Y |
| Beijing Children’s Hospital of Capital Medical University | Children' hospital | O | — | Y | 40 | 60 | 40 | 73 | Y | Y | Y | Y | Y | Y |
| The Affiliated Shenzhen Maternity and Child Healthcare Hospital of Southern Mediacal University | Perinatal center | I/O | 19793 | Y | 60 | 50 | 13 | 54 | Y | N | Y | N | N | Y |
| Jiangxi Provincial Children’s Hospital | Children' hospital | O | — | N | 75 | 70 | 31 | 117 | N | Y | Y | Y | N | Y |
| The First Affiliated Hospital of Anhui Medical University | General hospital | I/O | 6000 | Y | 20 | 40 | 11 | 43 | N | Y | Y | N | N | Y |
| Guiyang Maternal and Child Health Care Hospital | Perinatal center | I/O | 15000 | N | 70 | 65 | 40 | 93 | N | Y | Y | N | N | Y |
| The Third Xiangya Hospital of Central South University | General hospital | I/O | 4000 | Y | 15 | 25 | 6 | 36 | N | Y | Y | N | N | Y |
| Suzhou Municipal Hospital | General hospital | I/O | 18000 | Y | 41 | 70 | 22 | 76 | Y | N | N | N | N | Y |
| Children’s Hospital of Fudan University | Children' hospital | O | — | Y | 80 | 150 | 33 | 143 | Y | Y | Y | Y | Y | Y |
