# Supplementary material for: Mortality and Morbidity in Infants <34 Weeks' Gestation in 25 NICUs in China: A Prospective Cohort Study
Source: Front Pediatr. 2020 Feb 13;8:33. doi: 10.3389/fped.2020.00033 (PMC7031923; doi:10.3389/fped.2020.00033)
Supplement: Supplementary file 2 [file Table_2.docx]

**Supplementary Table 2 Maternal and Infant Characteristics of Inborn Infants Born at <34 Weeks’ Gestation**

| **Characteristics** | **All Infants** | **Complete Care Infants** | **DAMA Infants** | **P Value** |
| --- | --- | --- | --- | --- |
| Number of Infants | 5517 | 4735 | 782 | - |
| **Maternal Characteristics** |  |  |  |  |
| Prenatal care, n/N (%) | 5429 (98.8%) | 4668 (99.0%) | 761 (97.6%) | <0.01 |
| Maternal hypertension, n/N (%) | 896 (16.4%) | 769 (16.4%) | 127 (16.4%) | 0.97 |
| Maternal diabetes, n/N (%) | 636 (11.6%) | 572 (12.2%) | 64 (8.3%) | <0.01 |
| Antenatal steroids, n/N (%) | 3738 (68.5%) | 3252 (69.5%) | 486 (62.9%) | <0.01 |
| Primigravida, n/N (%) | 2175 (39.4%) | 1906 (40.3%) | 269 (34.4%) | <0.01 |
| Caesarean section, n/N (%) | 3116 (56.5%) | 2782 (58.8%) | 334 (42.7%) | <0.01 |
| **Infant Characteristics** |  |  |  |  |
| Gestational age (weeks), mean (SD) | 31.5 (2.0) | 31.6 (1.8) | 30.5 (2.3) | <0.01 |
| <26^+0^ weeks’, n/N (%) | 64 (1.2) | 37 (0.8) | 27 (3.5) |  |
| 26^+0^-26^＋6^ weeks’, n/N (%) | 95 (1.7) | 65 (1.4) | 30 (3.8) |  |
| 27^+0^-27^＋6^ weeks’, n/N (%) | 162 (2.9) | 103 (2.2) | 59 (7.5) |  |
| 28^+0^-28^＋6^ weeks’, n/N (%) | 338 (6.1) | 269 (5.7) | 69 (8.8) |  |
| 29^+0^-29^＋6^ weeks’, n/N (%) | 473 (8.6) | 380 (8) | 93 (11.9) |  |
| 30^+0^-30^＋6^ weeks’, n/N (%) | 662 (12) | 544 (11.5) | 118 (15.1) |  |
| 31^+0^-31^＋6^ weeks’, n/N (%) | 914 (16.6) | 795 (16.8) | 119 (15.2) |  |
| 32^+0^-32^＋6^ weeks’, n/N (%) | 1273 (23.1) | 1136 (24) | 137 (17.5) |  |
| 33^+0^-33^＋6^ weeks’, n/N (%) | 1536 (27.8) | 1406 (29.7) | 130 (16.6) |  |
| Birth weight (grams), mean (SD) | 1644 (407) | 1676 (398) | 1453 (411) | <0.01 |
| <750 grams, n/N (%) | 40 (0.7) | 25 (0.5) | 15 (1.9) |  |
| 750-999 grams, n/N (%) | 239 (4.3) | 157 (3.3) | 82 (10.5) |  |
| 1000-1249 grams, n/N (%) | 667 (12.1) | 513 (10.8) | 154 (19.7) |  |
| 1250-1499 grams, n/N (%) | 1029 (18.7) | 855 (18.1) | 174 (22.3) |  |
| 1500-1999 grams, n/N (%) | 2410 (43.7) | 2139 (45.2) | 271 (34.7) |  |
| ≥ 2000 grams, n/N (%) | 1132 (20.5) | 1046 (22.1) | 86 (11.0) |  |
| Male, n/N (%) | 3164 (57.4%) | 2742 (57.9%) | 422 (54.0%) | 0.04 |
| SGA, n/N (%) | 662 (12.0%) | 539 (11.4%) | 123 (15.7%) | <0.01 |
| 1-min Apgar≤3, n/N (%) | 223 (4.0%) | 152 (3.2%) | 71 (9.1%) | <0.01 |
| 5-min Apgar≤3, n/N (%) | 41 (0.7%) | 27 (0.6%) | 14 (1.8%) | <0.01 |
| TRIPS score, median (IQR) | 8.0 (6.0, 19.0) | 8.0 (5.0, 17.0) | 15.0 (8.0, 22.0) | <0.01 |

**Supplementary Table 3 Outcome Rates of Inborn Infants Born at <34 Weeks’ Gestation and Receiving Complete Care by Gestational Age**

| **Outcomes** | **<26^0^ weeks’** | **26^0^ to 27^6^ weeks’** | **28^0^ to 31^6^ weeks’** | **32^0^ to 33^6^ weeks’** | **Total** |
| --- | --- | --- | --- | --- | --- |
| Composite Outcome^a^, n/N (%) | 32/37 (86.5) | 114/168 (67.9) | 685/1988 (34.5) | 382/2542 (15.0) | 1213/4735 (25.6) |
| Mortality, n/N (%) | 17/37 (45.9) | 36/168 (21.4) | 79/1988 (4.0) | 23/2542 (0.9) | 155/4735 (3.3) |
| Sepsis^b^, n/N (%) | 11/37 (29.7) | 53/168 (31.6) | 397/1988 (20.0) | 199/2542 (7.8) | 660/4735 (13.9) |
| NEC^c^, n/N (%) | 1/26 (3.8) | 8/144 (5.6) | 69/1920 (3.6) | 39/2435 (1.6) | 117/4525 (2.6) |
| IVH or PVL^d^, n/N (%) | 6/15 (40.0) | 20/84 (23.8) | 93/1196 (7.8) | 58/1739 (3.3) | 177/3034 (5.8) |
| ROP^e^, n/N (%) | 5/15 (33.3) | 8/118 (6.8) | 13/1247 (1.0) | 2/719 (0.3) | 28/2099 (1.3) |
| BPD^f^, n/N (%) | 28/37 (75.7) | 65/168 (38.7) | 213/1988 (10.7) | 94/2542 (3.7) | 400/4735 (8.5) |
