# Supplementary material for: Mortality and Morbidity in Infants <34 Weeks' Gestation in 25 NICUs in China: A Prospective Cohort Study
Source: Front Pediatr. 2020 Feb 13;8:33. doi: 10.3389/fped.2020.00033 (PMC7031923; doi:10.3389/fped.2020.00033)
Supplement: Supplementary file 3 [file Table_3.docx]

**Supplementary Table 3 Outcome Rates of Inborn Infants Born at <34 Weeks’ Gestation and Receiving Complete Care by Gestational Age**

| **Outcomes** | **<26^0^ weeks’** | **26^0^ to 27^6^ weeks’** | **28^0^ to 31^6^ weeks’** | **32^0^ to 33^6^ weeks’** | **Total** |
| --- | --- | --- | --- | --- | --- |
| Composite Outcome, n/N (%) | 32/37 (86.5) | 114/168 (67.9) | 685/1988 (34.5) | 382/2542 (15.0) | 1213/4735 (25.6) |
| Mortality, n/N (%) | 17/37 (45.9) | 36/168 (21.4) | 79/1988 (4.0) | 23/2542 (0.9) | 155/4735 (3.3) |
| Sepsis, n/N (%) | 11/37 (29.7) | 53/168 (31.6) | 397/1988 (20.0) | 199/2542 (7.8) | 660/4735 (13.9) |
| NEC^a^, n/N (%) | 1/26 (3.8) | 8/144 (5.6) | 69/1920 (3.6) | 39/2435 (1.6) | 117/4525 (2.6) |
| IVH or PVL^b^, n/N (%) | 6/15 (40.0) | 20/84 (23.8) | 93/1196 (7.8) | 58/1739 (3.3) | 177/3034 (5.8) |
| ROP^c^, n/N (%) | 5/15 (33.3) | 8/118 (6.8) | 13/1247 (1.0) | 2/719 (0.3) | 28/2099 (1.3) |
| BPD, n/N (%) | 28/37 (75.7) | 65/168 (38.7) | 213/1988 (10.7) | 94/2542 (3.7) | 400/4735 (8.5) |

^a^Incidence of NEC ≥ stage 2 = number of infants with NEC ≥stage 2/ number of infants survived more than 72 hours

^b^Incidence of IVH ≥grade 3 or PVL= number of infants with IVH ≥grade 3 or PVL/ number of infants with neuroimaging results

^c^Incidence of ROP ≥ stage 3 = number of infants with ROP ≥stage 3/number of infants with eye examinations in NICU

Abbreviations: DAMA, infants discharged against medical advice; BPD, bronchopulmonary dysplasia; NEC, necrotizing enterocolitis; IVH, intraventricular hemorrhage; PVL, periventricular leucomalacia; ROP, retinopathy of prematurity
